# A novel subtilisin-producing *Bacillus velezensis* strain (BV-OLS1101) improves growth and mucosal immunity in broiler chickens under *Clostridium perfringens* challenge

**DOI:** 10.1016/j.psj.2025.105721

**Published:** 2025-08-24

**Authors:** S. Haldar, A.K. Dhara, I. Sengupta, S. Paul, S.S. Arora, R. Kumar, A. Pal, V. Kulkarni

**Affiliations:** a Agrivet Research and Advisory Pvt Ltd., 714 Block A Lake Town, Kolkata 700089, India; b Division of Pathophysiology, ICMR- National Institute for Research in Bacterial Infections (NIRBI), Kolkata 700010, India; c Optima Life Sciences Private Limited, Pune Maharashtra, 411009, India

**Keywords:** Bacillus velezensis, Subtilisin, Broiler chickens, Antibiotic alternative, Clostridium perfringens challenge

## Abstract

Antibiotic growth promoters (AGPs) are increasingly subject to global regulatory restrictions and consumer pressure, driving the poultry industry toward antibiotic-free production systems. This shift has accelerated the search for effective alternatives, including innovative microbial additives, organic acids, phytogenics, and other bioactive compounds capable of supporting digestive function and enhancing immune competence in poultry. The present study reported the isolation and characterization of a novel *Bacillus velezensis* strain, BV-OLS1101, possessing robust probiotic attributes and a distinctive capacity to produce a serine protease subtilisin. It was hypothesised that as a direct-fed microbial (DFM) BV-OLS1101, which was isolated from the soil of poultry farm and taxonomically identified by 16 s rRNA sequencing, might improve performance and mucosal immune status in broiler chickens particularly when the birds were enterically challenged with *Clostridium perfringens* (CP). BV-OLS1101 exhibited high thermotolerance (89 % survival at 80 °C), strong acid and bile resistance, broad antibiotic susceptibility, and *in vitro* antagonism against CP. Subtilisin production was confirmed through protease activity assays, phenyl methyl sulfonyl fluoride inhibition, and SDS-PAGE analysis. A 42-day *in vivo* trial was conducted using 432 male Ross 308 broilers randomly assigned to six groups in a 2 × 3 factorial design: unchallenged or CP-challenged birds, each with or without BV-OLS 1101 at 250 or 500 mg/kg feed (equivalent to 6 and 12 × 10^8^ CFU/g of feed respectively as a premix). Birds were fed an AGP-free maize - soybean meal diet. *Clostridium* challenge (10⁹ CFU/bird/day) was induced for 3 consecutive days starting from 18 days of age, following a coccidia priming on 14 days of age. Growth performance was assessed at weekly intervals, while intestinal lesion scores and the expression of cytokines and mucosal markers were assessed in birds at 28 days of age. In comparison to the unchallenged birds, CP challenge impaired feed conversion ratio (FCR) by 8.26 % (*P* = 0.005). Supplementation with BV-OLS1101 improved FCR by up to 7.9 % (*P* = 0.01) compared to the challenged birds. BV-OLS1101 reduced cecal and ileal lesions (*P* < 0.05), downregulated expression of IL-1β, IL-6, IFN-γ, and upregulated that of TGF-β, MUC2, and sIgA. Based on the present findings, subtilisin is proposed as a key immunomodulatory factor, supported by its observed expression and literature-documented bioactivity. Therefore, BV-OLS1101 may be considered a promising next-generation probiotic, which, by virtue of its heat stability and capacity to produce subtilisin, presents a novel biological strategy to reduce reliance on AGPs while sustaining gut health and improving performance in broiler chickens.

## Introduction

Probiotics derived from *Bacillus* spp. are increasingly incorporated into poultry feed formulations due to their ubiquitous presence in nature, resilience during feed processing and storage, and ability to regain viability in the gastrointestinal tract (**GIT**), where they germinate and proliferate under anoxic conditions (Ramlucken et al., 2020; Cartman et al., 2008; Chaiyawan et al., 2015; Nguyen et al., 2015). The renewed focus on *Bacillus*-based probiotics is largely driven by growing global concern over antimicrobial resistance (**AMR**), as reported by Sachdeva et al. (2024).The widespread use of AGPs in feed to manage poultry diseases such as necrotic enteritis (**NE**), primarily caused by *Clostridium perfringens* (**CP)**, has been linked directly or indirectly to the development of AMR in humans. This is a genuine concern which warrants the urgent search for effective non-AGP alternatives for use in livestock and poultry (Sachdeva et al., 2024). *Bacillus subtilis* (BS) is one of the most extensively studied non-AGP alternatives, shown to enhance intestinal barrier integrity, support mucosal immunity, improve digestive enzyme secretion, and optimise nutrient metabolism (Zhang et al., 2021; Yaqoob et al., 2022). Probiotics based on BS stimulate production of secretory IgA (**sIgA**), strengthen mucosal defences (Ningish et al., 2023), and secrete antimicrobial peptides (**AMPs**) that suppress pathogenic and opportunistic bacteria (Kan et al., 2021; Oladokun et al., 2021). Apart from BS, several other *Bacillus* species, like *B. amyloliquefaciens* (**BA**) or *B. licheniformis*, have long been studied for their beneficial applications in animal nutrition (Zhang et al., 2021; Yaqoob et al., 2022). More recently, *Bacillus velezensis* (**BV**) has emerged as a promising candidate, attracting growing interest from researchers (Ruiz-García et al., 2005; Mullins et al., 2020). Genomic analyses reveal that BV shares over 99 % similarity with both BS and BA (Wang et al., 2008), and it has been formally recognized as safe for feed use by regulatory bodies such as the European Food Safety Authority and the U.S. Food and Drug Administration (Quach et al., 2021). The reported probiotic potential of BV in poultry (Cui et al., 2024; Zhu et al., 2024) is likely mediated through its ability to disrupt pathogenic quorum sensing via the AMPs produced by this bacterium (Leistikow et al., 2024). As such BV is regarded as one of the most prolific AMP producers amongst different *Bacillus* spp., allocating approximately 4 % more of its genome to AMP biosynthesis compared to BS (Chen et al., 2006; Jin et al., 2025). Subtilisin is one of the noteworthy AMPs synthesised by BV. Subtilisin refers to a class of stable, extracellular serine proteases with broad substrate specificity, which contribute to plant defence (Hu et al., 2022) and have demonstrated anticancer activity (Singh et al., 2022). The multifunctionality and well-established safety profile of subtilisin indicates towards its potential as a viable alternative to AGPs in poultry nutrition (Shettar et al., 2023). In the present experiment, the probiotic potential of a novel subtilisin-producing BV (*Bacillus velezensis* strain OLS1101) was evaluated following its formulation as a direct-fed microbial (**DFM**). The basic objective of this experiment was to investigate the effects of BV-OLS1101 on growth performance and host immune responses in broiler chickens, with immune function evaluated through the expression of selected key immunomodulatory genes. The study was conducted under conditions of enteric stress induced by a controlled CP infection model designed to mimic subclinical NE, providing a relevant context to examine the functional role of BV-OLS1101 as a non-AGP intervention.

## Materials and methods

### Isolation, characterization and molecular identification of the bacterial strain

The bacterium was isolated from soil collected from a poultry farm in West Bengal, India. The process involved growing the bacterial culture in Luria-Bertani (**LB**) broth at 37 °C for 24 h in a shaker incubator at 180 rpm. The resulting culture was streaked onto LB agar, *Bacillus* Agar Base (**BAB**), and *Bacillus* Differential Agar (**BDA**) plates, followed by incubation at 37 °C for 24 h. Based on distinct colony morphology, a presumptive *Bacillus* isolate was selected which was designated as strain OLS 1101. Molecular identification was performed via 16S rRNA gene sequencing. Genomic DNA was extracted using a commercial DNA isolation kit (NucleoSpin® Microbial DNA, Takara Bio. The 16S rRNA gene was amplified using primers 27F (5′-AGAGTTTGATCCTGGCTCAG-3′) and 1492R (5′-GGTTACCTTGTTACGACTT-3′). Sequence similarity was assessed using the NCBI BLASTN tool . Phylogenetic lineage analysis was determined through neighbour-joining analysis with 1,000 bootstrap replications using MEGA 11 software (Sengupta and Dhal, 2024). The isolate was screened for key probiotic attributes, including hemolysin assay (Maragkoudakis et al., 2009), heat tolerance at 80 °C for 60 min (Zhu et al., 2024) and the ability of heat-resistant spores to revert to vegetative cells at 37 °C. Survival was also assessed under varying pH conditions (2, 4, 6, and 7.4) and bile salt concentrations (0.1 % and 0.2 %) for 2 h at 37 °C (Thirabunyanon et al., 2009). Antibiotic susceptibility was tested against levofloxacin (LE, 5 μg), doxycycline (DO, 30 μg), colistin (CL, 10 μg), amikacin (AK, 10 μg), neomycin (N, 30 μg), and amoxicillin (AMX, 10 μg) (Sarker et al., 2010). Antimicrobial activity was also evaluated against *Clostridium perfringens* MTCC 450 (Karimi et al., 2018).

### Evaluation of protease activity and subtilisin production

Protease activity of the bacterial isolate was confirmed using a skim milk agar (SMA) diffusion assay (Zhu et al., 2024). For further evaluation, the isolate was revived in nutrient broth and LB at 37 °C, and the culture supernatant was subjected to azocasein assay to quantify extracellular protease activity (Singh et al., 2022). Subtilisin characterization involved anion-exchange followed by size-exclusion chromatography, with eluates analyzed by SDS-PAGE (Singh et al., 2022).

### Animal experiment

The study was conducted at Agrivet Research and Advisory Pvt. Ltd., Kolkata, India, following ethical approval from the Institutional Animal Ethics Committee (Approval No. ARAPL/IAEC/011/24-25) in accordance with Committee for the Purpose of Control and Supervision of Experiments on Animals CPCSEA GUIDELINES FOR POULTRY/ BIRDS FACILITY Government of India Ministry of Fisheries (2020).

### Probiotic premix

The bacterial isolate of *B. velezensis* OLS1101 (BV-OLS1101) isolated from soil collected from a poultry farm was formulated into a prototype probiotic by Optima Life Sciences (Pune, India) following guidelines from Zhu et al. (2024). Briefly, the isolate was cultured in sporulation medium, mixed with skim milk powder and trehalose as protectants, freeze-dried, and milled into a powder for storage and use. The final probiotic formulation was incorporated into broiler diets at target concentrations of 6 and 12 × 10⁸ CFU/g feed. These levels exceed those commonly reported in the literature, which range from 1 × 10⁶ to 8 × 10⁷ CFU/g feed (Liu et al., 2021; Abudabos et al., 2020; Knap et al., 2010; Musa et al., 2019). However, this overage was intentional to ensure delivery of an effective dose to the birds, as probiotic cell loss during feed processing is well-documented and cannot be entirely avoided (Tripathi and Giri, 2014; Haffner et al., 2016; Ramlucken et al., 2021).

### Dietary treatments, bird husbandry, and induction of Clostridium challenge

The trial employed a maize - soybean meal-based diet formulated according to Ross 308 AP95 requirements (Aviagen, 2019). Dietary treatments included a non-challenged control (NC) and NC supplemented with BV-OLS1101 at 250 or 500 mg/kg feed (NC+BV-250 and NC+BV-500), delivering a minimum of 6 × 10⁸ and 12 × 10⁸ CFU/g feed, respectively. A challenged control (CC) group received the same diet, with or without BV supplementation (CC+BV-250 and CC+BV-500), and was subjected to CP challenge. The feeding trial of 42 days duration was conducted with a flock of 432 one-day-old male Ross 308 AP95 chickens procured from a commercial hatchery. There were 6 replicate pens per treatment, and each pen had 12 chicks at the beginning (*n* = 72 birds). The chicks were weighed (mean initial BW was 46 g) immediately after their arrival at the research farm and were distributed into four treatment groups according to a randomized block design with the absence or presence of infection serving as the blocking factor. The chicks were placed on litter (composed of wood shavings) in pens (1.2 m x 1.2 m). The experimental house was equipped with electrical brooding apparatus, tunnel ventilation, and evaporative cooling pads. Photoperiod was set at a ratio of 23 h light: 1 h dark for the first 7 days and then at 20 h light: 4 h dark till the birds were harvested. The temperature was set at 32°C for the first 7 days and 26°C for the remainder period of the study. Prior to the experiment, the water lines were decontaminated with 10 % bleach and flushed with fresh water to sanitize any possible contaminants. A three-phase feeding was practiced in which the starter (1-14 d) diet was given as crumbles and the grower (15-28 d), and the finisher (25-42 d) diets were given as pellets. The diets were prepared in a prototype pellet mill under a steam pressure of 2.5 kg/cm2, conditioning temperature of 80°C ± 2°C and a dwelling time of 40-50 seconds. All diets contained a phytase, which was used according to the full matrix suggested by the manufacturer (Quantum Blue 5 G, AB Vista Feed Ingredients, Marlborough, United Kingdom). No other gut-acting growth promoters, like non-starch polysaccharide degrading enzymes or proteases, were used in diets to avoid any possible confounding effects. No anticoccidials were used due to planned coccidial priming (Table 1). The birds received feed within 12 h of hatching. Manually operated feeders and nipple drinkers were fitted to each pen, and the birds had ad libitum access to both feed and water throughout the experiment. Vaccination involved that against infectious bronchitis (0 d), Newcastle disease (5 d and 20 d), and infectious bursal disease (12 d).

**Table 1:** Ingredients and calculated nutrient composition of the basal diets.

	Starter (1-14 d)	Grower (15-28 d)	Finisher (29-42 d)
Ingredients g/kg as fed			
Maize	576.66	632.46	656.2
Soybean meal^1^	348.14	302.36	274.64
Rice bran oil	33.7	32.07	42.19
Limestone powder	14.09	10.26	8.77
Mono calcium phosphate	11.7	8.17	5.51
DL-Methionine	3.97	3.53	3.18
L-Lysine HCl	2.83	2.52	2.14
L-Threonine	1.67	1.96	1.07
L-valine	0.5	0.35	0.3
L-Arginine	0.19	0.22	0.1
Salt	2.74	2.2	2.0
Sodium-bi-carbonate	2.0	2.0	2.0
Choline chloride	0.71	0.8	0.8
Vitamin premix^2^	0.5	0.5	0.5
Trace mineral premix^3^	0.5	0.5	0.5
*E. coli* phytase^4^	0.1	0.1	0.1
Nutrient composition (g/100 g as fed, unless stated otherwise)			
Dry matter*	89.02	88.89	88.98
Organic matter*	95.75	95.49	95.71
AME kcal/kg	2975	3050	3150
Crude protein *	22.41	20.52	18.62
Digestible amino acids			
Lysine	1.32	1.18	1.08
Methionine	0.70	0.64	0.59
Methionine + cysteine	1.00	0.92	0.86
Threonine	0.88	0.85	0.72
Tryptophan	0.24	0.21	0.20
Arginine	1.40	1.27	1.17
Isoleucine	0.88	0.80	0.75
Valine	1.00	0.91	0.84
Leucine	1.68	1.57	1.49
Histidine	0.54	0.49	0.46
Phenylalanine	1.00	0.92	0.86
Glycine	0.58	0.50	0.45
Serine	0.69	0.60	0.54
Ether extract *	5.92	6.0	7.06
Crude fibre *	2.7	2.67	2.63
Calcium *	0.82	0.73	0.62
Total phosphorus *	0.72	0.62	0.52
Available phosphorus	0.50	0.42	0.36
Phytate phosphorus	0.26	0.26	0.25
Sodium	0.22	0.2	0.19
Chloride	0.22	0.19	0.17
Potassium	0.98	0.9	0.85
Choline mg/kg	1700	1642	1579

1 Actual crude protein content was 48.6 %.

2 Contained (per kg) vitamin E 100 g, vitamin A 26000000 IU; vitamin D3 8000000 IU, pantothenic acid 30 g, vitamin K3 6 g, vitamin B1 6 g, vitamin B2 18 g, vitamin B6 10 g, vitamin B12 0.044 g, biotin 0.3 g, folic acid 4 g, niacin 120 g. (Rovimix Poultry Feed Supplement, DSM Nutritional Products India Ltd.).

3 Contained (per kg) manganese 60 g as yeast protein chelate, zinc 60 g as yeast protein chelate, iron 30 g as yeast protein chelate, copper 10 g as yeast protein chelate, selenium 0.6 g as yeast protein chelate, iodine 4 g as sodium iodide; chromium 1 g as yeast protein chelate (Organomin Forte, Zeus Biotech, India).

4 Declared phytase activity 5000 FTU/g (Quantum Blue, AB Vista, Marlborough, UK).

⁎ Analysed values.

*Clostridium* challenge was induced in all CC groups following in-house protocols (van der Klein et al., 2024). Birds were orally administered 6000 sporulated oocysts/bird/day of *Eimeria* spp. (*E. tenella, E. maxima, E. acervulina*) at day 14 (Haug et al., 2007), followed by oral inoculation with *Clostridium perfringens* MTCC 450 (10⁹ CFU/bird/day) on 3 consecutive days from 18-d of age. The CP culture was freshly prepared from stock maintained at Agrivet Laboratories.

Body weight (BW) was recorded on a replicate basis at weekly intervals at 0800 h without prior fasting. Data for 21-d and 42-d were reported to correspond with the completion of the challenge and the end of the trial, respectively. Average daily gain (ADG) was calculated for 1 to 21- d (pre-challenge) and 22 to 42-d (post-challenge). Feed was offered daily in two equal portions, and cumulative feed intake (FI) was recorded weekly. Average daily feed intake (ADFI) was calculated for 1 to 21, 22 to 42, and 1 to 42-d periods by subtracting residual feed from the total offered. Feed conversion ratio (FCR) was computed as the ratio of ADFI to ADG for each period. Mortality and culling were recorded daily; liveability was reported for the pre-challenge period (1 to 21-d), weekly during post-challenge (22 to 42-d), and cumulatively for the entire 42-d trial.

### Euthanasia of birds and small intestinal lesion scoring

On 28-d of age, two birds per pen were euthanized by cervical dislocation and eviscerated. The small intestine was excised, and digesta was expelled by gentle pressure. The tissue was rinsed with phosphate-buffered saline (**PBS**) to remove debris and blood. A longitudinal incision was made to open the lumen, and lesions were scored for dysbacteriosis and coccidiosis following the methods of de Gussem (2010) and Johnson and Reid (1970), with minor modifications in the number of birds evaluated. Dysbacteriosis scoring employed a binary system (0 = no lesion, 1 = lesion) to evaluate parameters such as ballooning of the GIT, visible inflammation, reduced tonus, wall fragility, and abnormal contents in both the cranial and caudal intestine (divided at Meckel’s diverticulum). The presence of undigested feed beyond the ileocecal junction was assessed as an additional parameter. Scores were averaged to obtain the Mean Bacterial Enteritis Score (**MBES**). Coccidial lesions were scored individually for *E. maxima, E. acervulina*, and *E. tenella* on a 0 - 4 scale, and summed to calculate the Total Mean Lesion Score (**TMLS**) (Fig. 4).

### Collection of mucosal scrapping and internal organs and transcriptomic analyses

The small intestine was divided into two segments, with the duodenum and jejunum extending from the duodenal loop to Meckel’s diverticulum, and the ileum extending from Meckel’s diverticulum to the ileo-cecal junction. Mucosal scrapings were collected from both segments of the small intestine of all birds euthanized on day 28 and pooled pen-wise using a microscalpel, transferred into sterile tubes, and stored at - 80 °C until analysis. From these samples, the analysis of secretory IgA (sIgA) and transcriptomic profiling of MUC2, a gene crucial for maintaining mucosal integrity, was performed. Approximately 100 mg tissue samples from the spleen and liver were also collected post-wash with PBS for transcriptomic analysis of interleukin 1β and 6 (IL-1β, IL-6), interferon γ (IFN-γ), tumor necrosis factor-α (TNF-α), and transforming growth factor-β (TGF-β) (Haldar et al., 2025). Transcriptomic analyses followed the method of Haldar et al. (2025), using GAPDH as the housekeeping gene. Primers (Table 2) for target genes were designed using Primer3 Plus. Gene expression was quantified as both cycle threshold (Ct) and fold-change (2^–ΔΔCt^) relative to the NC, which was assigned a baseline value of 1, which was equivalent to 100 % of expression (Livak and Schmittgen, 2001). For each biological replicate, the mean of three technical replicates was used as a single data point.

**Table 2:** List of primers used for mRNA expression analysis.

Genes	Accession number	Size (bp)	Forward Primer (5′–3′)	Reverse Primer (5′–3′)
IL-6	AJ309540	254	CAAGGTGACGGAGGAGGAC	TGGCGAGGAGGGATTTCT
IL-1 β	Y15006	244	TGGGCATCAAGGGCTACA	TCAAAGGCCAAGAACATTCC
TNF α	HQ739087.1	162	GACCAGATGGGAAGGGAATG	TCACGATCATCTGGTTACAG
IFN-γ	X99774.1	151	AGCTCCCGATGAACGAC	CAGGAGGTCATAAGATGCCA
TGF-β	M31160.1	296	GGACGGATGAGAAGAACTG	ACGGACCACCATATTGGA
MUC-2	JX284122.1	94	CAGCGTTAACACAGGGCTTA	GCAGCAGACGTTGATCTCAT
sIgA	S40610	192	GTCACCGTCACCTGGACTACA	ACCGATGGTCTCCTTCACATC
GAPDH	NM_204305	144	GCAGATGCAGGTGCTGAGTA	GACACCCATCACAAACATGG

### Chemical analysis of diet

Representative feed samples from each dietary group were analysed for dry matter (DM; AOAC 934.01), crude protein (CP; AOAC 954.01), ether extract (EE; AOAC 920.39), crude fibre (CF; AOAC 962.09/978.10), ash (AOAC 942.05-2012), organic matter (OM; AOAC 942.05), calcium (AOAC 927.02-1990), and total phosphorus (AOAC 965.17-1966).

### Statistical analysis

Data were analysed using a 2 × 3 factorial design (Two-way ANOVA) in PROC GLM of SAS. The main effects included challenge (absence/presence) and BV-OLS1101inclusion level, along with their interaction. The statistical model used was: Y_ijk_ = μ + α_i_ + β_j_ + (α*β)_ij_ + ε_ijk_ where, Y_ijk_ is the response variable, μ is the overall mean, α_i_ is the effect of ith level of challenge, β_j_ is the effect of jth level of BV-OLS1101, and (α*β)_ij_ is the interaction effect between the challenge and BV-OLS1101 (referred to hereafter as “interaction effects”), and ε_ijk_ is the random error term. Main effects and the interaction effects are reported separately. Whenever a significant difference was detected, means were separated using Tukey’s B test. Significance was set at *P* < 0.05, and trends were noted for 0.05 ≤ *P* < 0.10. The distribution of residuals was assessed for normality using normal Q–Q plots and detrended Q–Q plots. Visual inspection confirmed that data points were reasonably aligned with the reference line, and deviations from normality, where present, were minor.

## Results

### Characterisation, identification and probiotic properties of BV-OLS1101

**Fig. 1:**
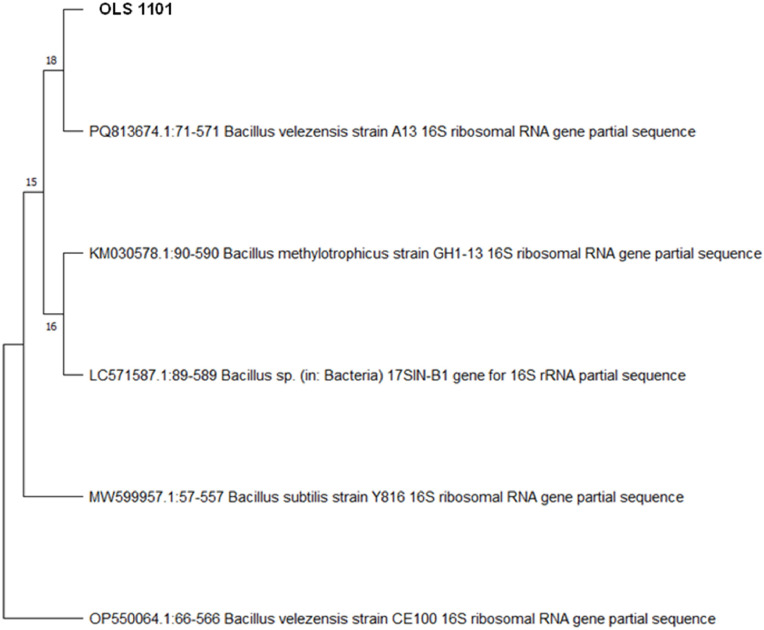
Phylogenetic lineage analysis of the bacterial isolate OLS 1101 showing closest affiliation with *Bacillus velezensis*.

**Fig. 2:**
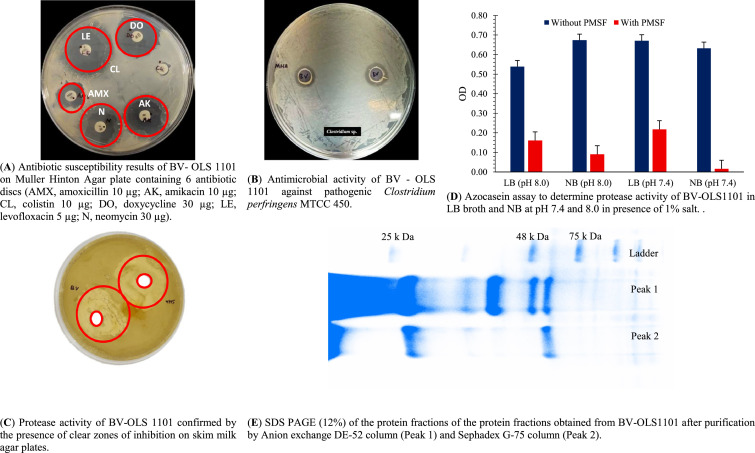
Evaluation of probiotic characters and confirmation of protease and subtilisin synthesising properties of *Bacillus velezensis* – OLS1101.

The morphology of BV-OLS1101 appeared white, circular, shelly, rough, and swarming in nature. Since the strain was cultivated on *Bacillus*-specific media (BAB and BDA), it was initially presumed to belong to the genus *Bacillus*. This was confirmed through molecular identification, and NCBI-BLAST analysis indicated high sequence similarity with *Bacillus velezensis*, a member of the phylum Firmicutes and family *Bacillaceae* (Fig. 1). Phylogenetic analysis further revealed that the isolate shared the closest homology with *B. velezensis* A13 (accession no. PQ813674.1) and accordingly, the isolate was identified as *Bacillus velezensis* OLS1101 (BV-OLS1101; accession no. PV643222). BV-OLS1101 did not show any α - or β - hemolysis in sheep blood agar media and hence appears to be free from any cytotoxic effects. The spores of BV-OLS1101 also exhibited high thermostability, with 89 % recovery following exposure to 80°C for 60 minutes. Subsequent culturing in LB medium confirmed vegetative outgrowth, yielding a viable count of 5 × 10⁸ CFU/ml. Under simulated acidic conditions, the strain retained 82.8 %, 93.4 %, 99.3 %, and 100 % viability after 2 h of incubation at pH 2.0, 4.0, 6.0, and 7.4, respectively. It also showed strong bile salt tolerance, maintaining 100 % and 97.4 % viability in the presence of 0.1 % and 0.2 % (w/v) bile salts, respectively. Antibiotic sensitivity testing via disc diffusion assay revealed that the strain was susceptible to all antibiotics tested, except for CL (Fig. 2A). In terms of antimicrobial activity, BV-OLS1101 inhibited the growth of *C. perfringens* MTCC 450 in vitro (Fig. 2B).

**Table:** 

Summary of the taxonomic affiliation of the 16S rRNA gene sequence-based BLAST search of OLS 1101					
Strain:	Phylum:	Class:	Family:	Genus:	Probable species:
OLS 1101	Firmicutes	Bacilli	*Bacillaceae*	*Bacillus*	*velezensis*

.

The appearance of clear zones around colonies of BV-OLS1101 on skimmed milk agar plates (Fig. 2C) indicated extracellular protease activity. This observation was corroborated by azocasein assays (Fig. 2D), which detected proteolytic activity in the culture supernatants of both LB and NB media. The marked reduction in enzymatic activity after addition of phenyl methyl sulfonyl fluoride (Fig. 2D), a specific serine protease inhibitor, suggested that the proteolytic function was primarily mediated by a serine protease. Sequential purification of the culture supernatant using anion-exchange chromatography followed by size-exclusion chromatography exhibited enhanced protease activity. SDS-PAGE analysis of the purified fraction revealed two distinct bands with approximate molecular weights of 29 kDa and 50 kDa (Fig. 2E). These sizes correspond to the mature and precursor forms of subtilisin-like serine proteases (Singh et al., 2022). Taken together, these findings confirm that BV-OLS1101 is a serine protease-producing *Bacillus* strain, with the capability to synthesize subtilisin-type antimicrobial peptides.

### Broiler performance

The performance traits of male broiler chickens during the pre-challenge, post-challenge, and overall (1-42 days) periods are presented in Table 3. Initial BW at day 0 was statistically similar across all treatment groups (*P* = 0.613), confirming uniform baseline conditions at the beginning of the trial.

**Table 3:** Effect of BV-OLS1101 and *Clostridium* challenge on performance during the pre-challenge (1-21 days) and post-challenge (22-42 days) periods: main effects and interactions^1^.

	Body weight g			ADG g				ADFI g			FCR			Periodic liveability %		
	0-d	21-d	42-d	1-21 d	22-42 d	1-42 d		1-21 d	22-42 d	1-42 d	1-21 d	22-42 d	1-42 d	1-21 d	22-42 d	1-42 d
Main effect: Challenge																
Absent	46.43	1167.1	3055.7	53.36	89.94	71.65		65.5	158.26	111.89	1.228	1.762	1.562	100.0	92.6	92.6
Present	46.37	1153.0	3001.3	52.7	88.01	70.36		63.73	162.77	113.25	1.209	1.853	1.611	99.2	90.6	88.4
P-value	0.46	0.171	0.129	0.174	0.226	0.129		0.018	0.099	0.35	0.102	0.005	0.008	0.21	0.5	0.176
Main effect: BV-OLS1101 (mg per kg diet)																
0	46.4	1163.3	2990.3	53.19	87.0	70.09	65.61		162.12	113.87	1.234	1.868	1.626	99.77	90.22	89.58
250	46.45	1158.3	3051.5	52.95	90.15	71.55	63.57		160.4	111.99	1.201	1.781	1.566	99.54	92.30	90.97
500	46.35	1158.6	3043.9	52.96	89.78	71.37	64.68		159.02	111.84	1.221	1.773	1.568	99.54	92.24	90.97
P-value	0.36	0.9	0.308	0.9	0.212	0.307	0.08		0.635	0.449	0.054	0.026	0.01	0.79	0.808	0.91
Interaction effect: Challenge*BV-OLS1101																
NC	46.35	1167.6	3042.6	53.39	89.28	71.34	65.8		159.48	112.64	1.233	1.788	1.579	100.0	93.06	93.06
NC+BV-250	46.58	1179	3077.8	53.93	90.42	72.17	65.34		157.81	111.58	1.212	1.747	1.547	100.0	93.06	93.06
NC+BV-500	46.38	1154.6	3046.9	52.77	90.11	71.44	65.37		157.5	111.44	1.239	1.75	1.561	100.0	91.67	91.67
CC	46.45	1159	2938	52.98	84.71	68.85	65.42		164.77	115.09	1.235	1.949	1.673	99.54	87.38	86.11
CC+BV-250	46.33	1137.6	3025.1	51.97	89.88	70.93	61.8		163	112.4	1.189	1.814	1.585	99.07	91.54	88.89
CC+BV-500	46.32	1162.5	3040.9	53.16	89.45	71.3	63.98		160.53	112.25	1.204	1.796	1.575	99.07	92.80	90.28
Pooled SEM	0.05	5.01	17.4	0.24	0.78	0.41	0.36		1.32	0.72	0.005	0.015	0.009	0.16	1.48	1.51
P-value	0.346	0.139	0.519	0.143	0.493	0.518	0.196		0.926	0.866	0.368	0.265	0.163	0.79	0.642	0.755

1 Means of 6 replicate pens per treatment (12 birds per pen at the beginning of the experiment). NC, non-challenged control; CC, challenged control; BV-OLS1101, Bacillus velezensis–based direct-fed microbial, supplemented at 250 mg/kg (BV-250) and 500 mg/kg (BV-500), corresponding to 6 × 10⁸ and 12 × 10⁸ CFU/g feed, respectively. SEM, standard error of the mean.

***Effects of CP challenge****:* The CP challenge did not exert a statistically significant effect on BW measured at either 21 days (*P* = 0.171) or 42 days (*P* = 0.129). ADG was not significantly affected by CP challenge during the pre-challenge (*P* = 0.174), post-challenge (*P* = 0.226), or overall periods (*P* = 0.129). ADFI was moderately influenced by challenge status. During the pre-challenge phase, ADFI was significantly lower in the CC+BV-250 group compared to the NC group (*P* = 0.018). The most pronounced impact of CP challenge was observed in FCR. Birds in the CC group exhibited significantly poorer FCR during the post-challenge (*P* = 0.005) and overall (1 - 42 days) period (*P* = 0.008). The challenge resulted in an 8.26 % deterioration in FCR during the post-challenge period and a 5.62 % decline over the full trial compared to the NC group. No significant difference in FCR was observed during the pre-challenge phase (*P* = 0.102).

***Effects of BV-*OLS1101*:*** Supplementation did not significantly influence ADG during the pre-challenge (*P* = 0.9), post-challenge (*P* = 0.212), or overall periods (*P* = 0.307). ADFI was not significantly affected by BV-DFM supplementation at any stage, though a trend toward lower ADFI could be observed in the CC+BV-250 group during the pre-challenge period (*P* = 0.080). Notably, FCR responded favourably to BV-DFM. While a trend toward improved FCR was evident during the pre-challenge period (*P* = 0.054), significant improvements were observed during both the post-challenge (*P* = 0.026) and overall (*P* = 0.01) periods.

***Interaction effects****:* No statistically significant CP challenge*BV-OLS1101 interactions were detected for BW at 21-d (*P* = 0.139) or 42-d (*P* = 0.519). Similarly, no significant interactions were observed for ADG during the pre-challenge (*P* = 0.143), post-challenge (*P* = 0.493), or overall (*P* = 0.518) phases. Although interaction effects on ADFI and FCR did not reach statistical significance (*P* > 0.05), substantial numerical improvements in FCR were evident in the CC+BV-250 and CC+BV-500 groups which showed 6.92 % and 7.85 % improved FCR, respectively, during the post-challenge period and 5.26 % and 5.85 % improvements over the 1 to 42-d period, compared to the CC group.

### Periodic liveability

During the pre-challenge period (1–21 d), liveability remained at or near 100 % across all treatment groups, with no mortality differences observed (Table 3).

***Effect of CP challenge*:** Coccidial priming at 14-d of age followed by CP challenge did not significantly affect flock liveability (*P* = 0.259). During the post-challenge period, weekly liveability trends revealed modest numerical differences among groups, although no statistically significant effects of CP challenge could be detected (*P* > 0.05). In the first week following the challenge (22 to 28-d), the CC group showed a non-significant decrease in liveability compared to the NC group (*P* = 0.675). From 29 to 35-d (*P* = 0.286) and 36 to 42-d (*P* = 0.99), liveability remained similar across all treatments, with differences narrowing further.

***Effect of BV-OLS1101*:** Liveability remained unaffected by BV-OLS1101 supplementation in both the pre-challenge (*P* = 0.176) and post-challenge phases (*P* > 0.05). However, birds in the CC+BV-250 group showed numerically higher survival than those in the CC group, while the CC+BV-500 group maintained liveability comparable with the NC group, indicating a mild dose-dependent trend associated with BV-OLS1101 in reducing post-challenge mortality. By the final interval (36 to 42-d), liveability was similar across all treatments (*P* = 0.825). Although weekly patterns hinted at a possible benefit of BV-OLS1101 supplementation, particularly at the 500 mg/kg inclusion level, in reducing early post-challenge mortality, these effects did not reach statistical significance.

***Interaction effects*:** No CP challenge*BV-OLS1101 interactions could be observed at any period of measurements with regard to periodic liveability of the experimental flock (*P* > 0.05). Overall cumulative survival remained high, and the differences among treatments were minimal.

### Small intestinal lesion scores

**Fig. 3:**
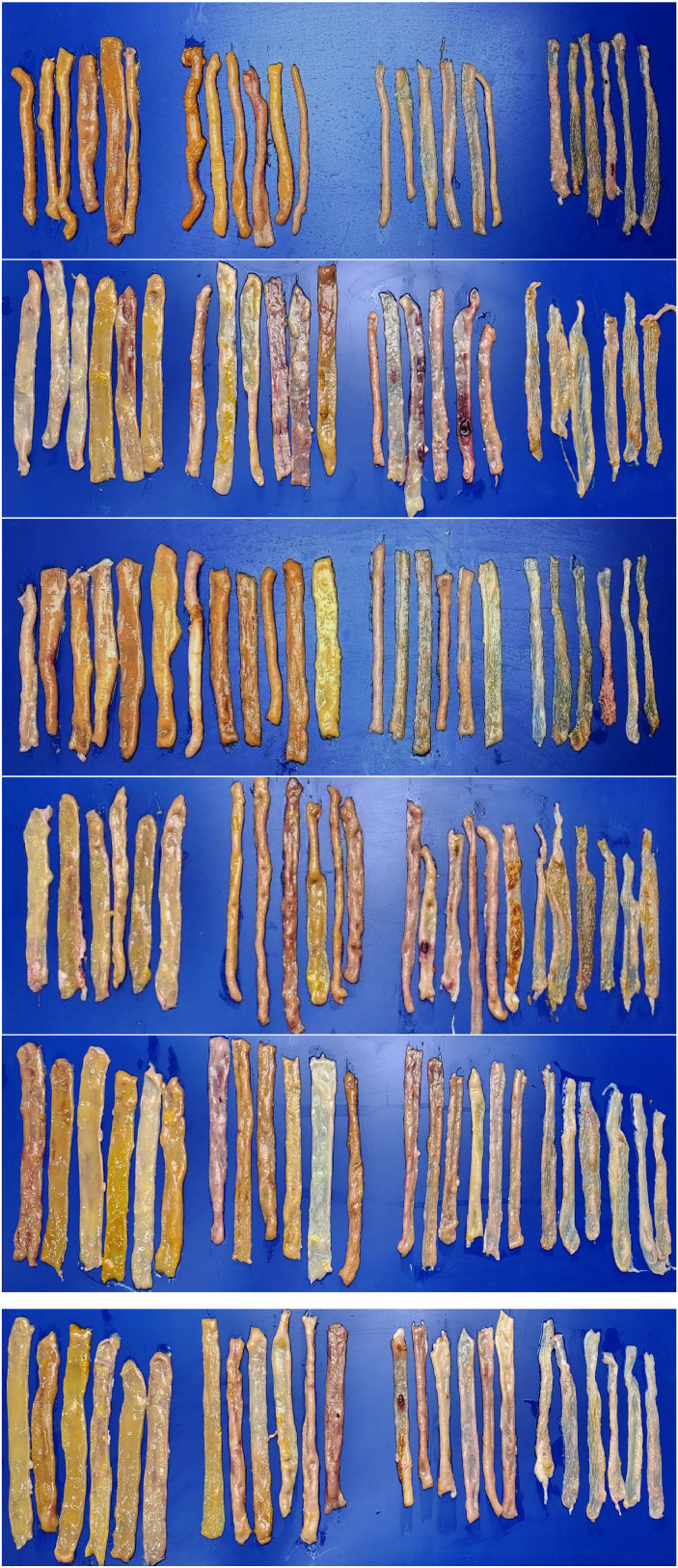
Small intestinal lesions determined on 21 days of age in experimental birds supplemented with BV-DFM and challenged with *Clostridium perfringens*.

**Fig. 4:**
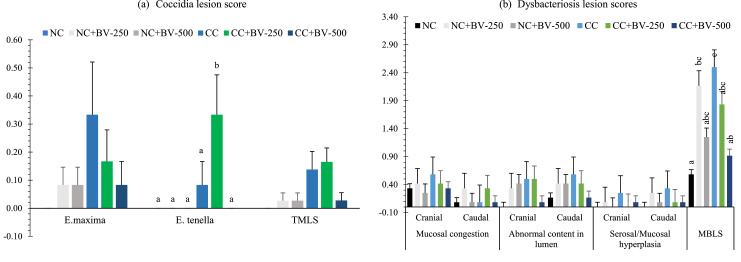
Small intestinal lesion scores determined on 21 days of age in experimental birds supplemented with BV-DFM and challenged with *Clostridium perfringens* (mean ± SE)^1^. ^1^*n* = 12 birds per treatment (two birds were randomly selected from each replicate pen). Each bar represents mean ± standard error of the mean (SEM). Bars with different letters indicate significant differences (*P* < 0.05). Bars with different letters indicate significant differences. TMLS, total mean lesion score; MBES, mean bacterial enteritis score. NC, non-challenged control; CC, challenged control; BV-DFM, *Bacillus velezensis-*based direct-fed microbial, supplemented at 250 mg/kg (BV-250) and 500 mg/kg (BV-500), corresponding to 6 × 10⁸ and 12 × 10⁸ CFU/g feed, respectively.

Photographic illustrations of small intestinal lesion assessment presented in Fig. 3 reveals that the NC group exhibited generally normal mucosal architecture with no visible signs of inflammation. In contrast, the CC group showed visible signs of mucosal thickening and congestion, suggestive of inflammatory responses likely to be caused by the CP challenge. Among the BV-OLS1101 supplemented groups, the CC+BV-250 birds displayed mild improvements, while the CC+BV-500 birds demonstrated clearer mucosal surfaces with reduced visual signs of damage compared to the CC. The visual findings are largely supported by the quantitative data presented in Fig. 4 (a and b) and the results are described below.

***Effects of CP challenge*:** Coccidia priming followed by CP challenge significantly elevated coccidial lesion scores, specifically for *E. tenella* (*P* = 0.014) and TMLS (*P* = 0.005), indicating a clear aggravation of pathological changes in the small intestine following the challenge (Fig. 4a). For dysbacteriosis-associated lesions assessed in cranial and caudal regions of the small intestine (Fig. 4b), CP challenge did not elicit significant changes in individual lesion parameters or in MBES (*P* > 0.05). Similarly, NE scores, evaluated based on mucosal damage and presence of necrotic foci, remained statistically unaltered across groups (*P* > 0.05).

***Effects of BV-OLS1101*:** Dietary inclusion of BV-OLS1101 decreased *E. tenella* lesion scores (*P* = 0.042), with the highest dose (500 mg/kg) showing the most prominent benefit. Supplementation also led to a significant reduction in MBES in the CC+BV-500 group (*P* = 0.007), suggesting that BV-DFM may alleviate bacterial enteritis-related damage under challenge conditions. Numerically lower scores were also noted in the CP-challenged birds receiving BV-DFM for parameters such as mucosal congestion, abnormal luminal content, and mucosal or serosal hyperplasia. NE lesion scores showed a downward trend in the BV-OLS1101 supplemented groups; however, differences did not reach statistical significance (*P* > 0.05), possibly due to low overall lesion severity and high within-group variation.

***Interaction effects*:** A significant CP challenge*BV-OLS1101 interaction was observed for *E. tenella* lesions (*P* = 0.042), with the CC+BV-500 group demonstrating the greatest reduction in lesion severity. A similar interaction was detected for MBES (*P* = 0.006), indicating that the efficacy of BV-OLS1101 in mitigating bacterial enteritis-like lesions was dependent on the presence of CP challenge. No significant interaction effects were observed for dysbacteriosis-related individual lesion scores or NE scoring (*P* > 0.05), though numerical trends suggested some degree of protective influence from BV-DFM, particularly at the higher inclusion level.

The photographic evidence in Fig. 3 corroborates the quantitative lesion scores in Fig. 4. The NC group displayed largely intact intestinal architecture, with only occasional haemorrhagic spots consistent with low *E. tenella* and TMLS values. In contrast, the CC group exhibited pronounced mucosal damage and haemorrhagic patches, corresponding to significantly higher *E. tenella* lesion scores and TMLS. Supplementation with BV-OLS1101 (250 and 500 mg/kg) visibly reduced lesion severity, which was reflected in significantly lower *E. tenella* scores and a significant interaction effect, particularly at 500 mg/kg. For dysbacteriosis-associated lesions, CC+BV-500 showed clear visual improvements, aligning with reduced MBES and supported by a significant interaction and a tendency toward a main effect of BV-OLS1101. Despite some within-group variability, the combined visual and quantitative evidence supports a protective role of BV-OLS1101 against CP-induced intestinal damage.

### Expression of pro-inflammatory and anti-inflammatory cytokines in intestine and other tissues

**Fig. 5:**
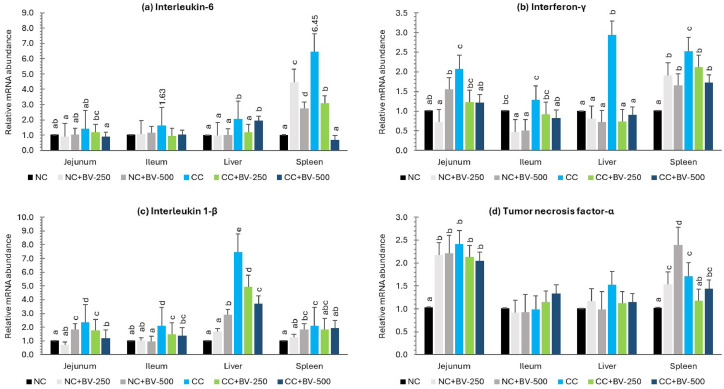
Effect of BV-DFM and *Clostridium* challenge on relative expression (2^-ΔΔCt^) of pro-inflammatory cytokines in small intestine, liver and spleen (28 days of age)^1^. ^1^*n* = 6 birds per treatment (one bird was randomly selected from each replicate pen). Each bar represents mean ± standard error of the mean (SEM). Bars with different letters indicate significant differences. NC, non-challenged control; CC, challenged control; BV-DFM, Bacillus velezensis–based direct-fed microbial, supplemented at 250 mg/kg (BV-250) and 500 mg/kg (BV-500), corresponding to 6 × 10⁸ and 12 × 10⁸ CFU/g feed, respectively.

**Fig. 6:**
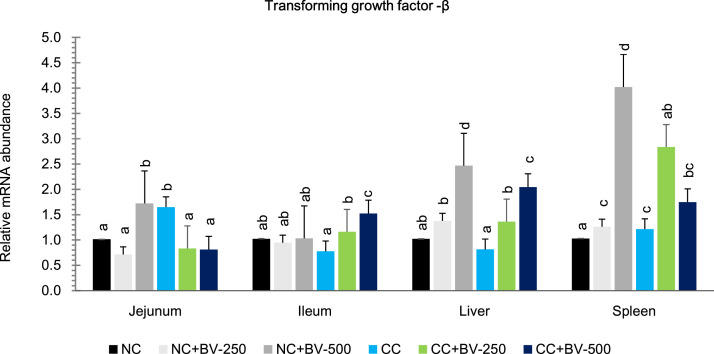
Effect of BV-DFM and *Clostridium* challenge on relative expression (2^-ΔΔCt^) of anti-inflammatory cytokine (TGF-β) in small intestine, liver and spleen (28 days of age)^1^.

**Fig. 7:**
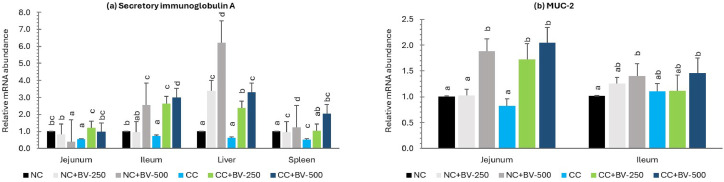
Effect of BV-DFM and *Clostridium* challenge on relative expression (2^-ΔΔCt^) of secretory immunoglobulin A and MUC2 gene in different tissues (28 days of age)^1^. ^1^*n* = 6 birds per treatment (one bird was randomly selected from each replicate pen). Each bar represents mean ± standard error of the mean (SEM). Bars with different letters indicate significant differences. NC, non-challenged control; CC, challenged control; BV-DFM, *Bacillus velezensis-*based direct-fed microbial, supplemented at 250 mg/kg (BV-250) and 500 mg/kg (BV-500), corresponding to 6 × 10⁸ and 12 × 10⁸ CFU/g feed, respectively.

The mRNA expression profiles of inflammatory cytokines IL-6 (Fig. 5a), IFN-γ (Fig. 5b), IL-1β (Fig. 5c), and TNF-α (Fig. 5d), and regulatory cytokine, TGF-β (Fig. 6) are presented graphically. Across all tissues examined, CP challenge markedly upregulated the expression of key pro-inflammatory cytokines, indicating both localized and systemic immune activation (Fig. 5a–d). Dietary supplementation with the BV-OLS1101 (6 × 10^8^ and 12 × 10^8^ CFU/ g Feed) consistently mitigated these inflammatory responses and enhanced the expression of the regulatory cytokine TGF-β (Fig. 6). Significant interaction effects further demonstrated that the immunomodulatory capacity of BV-DFM was most evident under challenge conditions, and the hypothesis gets corroboration from the expression pattern of immune-modulatory parameters, sIgA and MUC2 (Fig. 7a and b respectively).

**Table:** 

Foot notes to Fig. 5				
Fig. 5(a): Interleukin 6	Jejunum	Ileum	Liver	Spleen
Main effects:				
CP-challenge P =	0.002	0.409	0.0001	0.005
BV-DFM P =	0.002	0.181	0.0001	0.0001
CP challenge * BV-DFM *P*	0.002	0.06	0.0001	0.0001

Fig. 5(b): Interferon gamma (IFN -γ):				
Main effects:				
CP-challenge P =	0.001	0.0001	0.0001	0.0001
BV-DFM P =	0.001	0.0001	0.0001	0.022
CP challenge * BV-DFM *P*	0.0001	0.693	0.0001	0.0001

Fig. 5(c): Interleukin 1-β				
Main effects:				
CP-challenge P =	0.0001	0.0001	0.0001	0.001
BV-DFM P =	0.001	0.001	0.0001	0.203
CP challenge * BV-DFM *P*	0.0001	0.004	0.0001	0.057

Fig. 5(d): Tumor Necrosis Factor - α				
Main effects:				
CP-challenge P =	0.16	0.019	0.108	0.001
BV-DFM P =	0.358	0.434	0.447	0.0001
CP challenge * BV-DFM *P*	0.048	0.123	0.218	0.0001

Foot note to Fig. 6:				
Transforming growth factor Beta				
Main effects:				
CP-challenge P =	0.712	0.011	0.029	0.139
BV-DFM P =	0.005	0.0001	0.0001	0.0001
CP challenge * BV-DFM *P*	0.0001	0.0001	0.221	0.0001

Foot note to Fig. 7				
Fig. 7(a): Secretory immune globulin A				
Main effects:				
CP-challenge P =	0.02	0.0001	0.0001	0.109
BV-DFM P =	0.001	0.0001	0.0001	0.0001
CP challenge * BV-DFM *P*	0.0001	0.0001	0.0001	0.0001

Fig. 7(b): MUC 2				
Main effects:				
CP-challenge P =	0.095	0.999		
BV-DFM P =	0.0001	0.001		
CP challenge * BV-DFM *P*	0.029	0.364		

.

***Effects of CP challenge*:** In the jejunum, CP challenge significantly upregulated the expression of IL-1β (*P* < 0.0001), IL-6 (*P* = 0.002), and IFN-γ (*P* = 0.001) relative to the NC groups, whereas expression of TNF-α (*P* = 0.16) and TGF-β (*P* = 0.712) remained unaffected. In the ileum, CP challenge led to significant upregulation of IL-1β (*P* = 0.0001), IFN-γ (*P* = 0.0001), and TNF-α (*P* = 0.019), and a reduction in TGF-β expression (*P* = 0.011). IL-6 expression was not significantly altered (*P* = 0.409). In hepatic tissue, expression of IL-1β, IL-6, and IFN-γ (*P* = 0.0001 for all), as well as TGF-β (*P* = 0.029) was upregulated by CP challenge, while that of TNF-α remained unchanged (*P* = 0.108). In the spleen, systemic immune stimulation was evidenced by upregulated IL-1β (*P* = 0.001), IL-6 (*P* = 0.005), IFN-γ (*P* = 0.0001), and TNF-α (*P* = 0.001) expression. TGF-β expression in the spleen was not significantly influenced by CP challenge (*P* = 0.139).

***Effects of BV-OLS1101*:** Dietary supplementation of BV-OLS1101 particularly at 250 mg/kg diet significantly downregulated expression of IL-1β (*P* = 0.001), IL-6 (*P* = 0.002), and IFN-γ (*P* = 0.001) in jejunum. At 500 mg/kg BV-OLS1101 upregulated TGF-β (*P* = 0.005) expression in the same tissue. TNF-α expression was not significantly altered (*P* = 0.358). In the ileum, BV-OLS1101 supplementation downregulated IL-1β (*P* = 0.001), and IFN-γ (*P* = 0.0001), while significantly upregulating TGF-β (*P* = 0.0001); TNF-α (*P* = 0.434) and IL-6 (*P* = 0.181) remained unaffected. These changes were more pronounced in the CC groups, with a clear dose-dependent response, as the higher level of BV-OLS1101 supplementation elicited greater effects. In hepatic tissue, BV-OLS1101 supplementation resulted in a marked reduction in IL-1β, IL-6, and IFN-γ (*P* = 0.0001), along with a concurrent increase in TGF-β expression (*P* = 0.0001). The dose response, however, was not very conspicuous in this case. TNF-α expression was not significantly affected in the liver (*P* = 0.447). In the spleen, BV-OLS1101 significantly downregulated IL-6 (*P* = 0.0001), IFN-γ (*P* = 0.022), and TNF-α (*P* = 0.0001), and upregulated TGF-β (*P* = 0.0001).

***Interaction effects*:** In jejunum, significant CP challenge*BV-OLS1101 interactions for IL-1β (*P* = 0.0001, IL-6 (*P* = 0.002), IFN-γ (*P =* 0.002), TGF-β (*P* = 0.0001 for all), and TNF-α (*P* = 0.048), indicating an amplified cytokine-modulating effect of BV-DFM challenge conditions. In ileum, interactions were significant or tended to be significant for IL-1β (*P* = 0.004), TGF-β (*P* = 0.0001), and IL-6 (*P* = 0.06) but not for IFN-γ (*P* = 0.693) and TNF-α (*P* = 0.123). In the liver, interactions were evident for IL-1β, IL-6, and IFN-γ (*P* = 0.0001) only. In spleen, interactions were significant or tended to be significant for IL-6, IFN-γ, TNF-α, and TGF-β (*P* = 0.0001), and IL-1β (*P* = 0.057).

### Expression of sIgA and MUC2 in intestine and other tissues

Expression of mucosal immune effector sIgA (Fig. 7a) in jejunum, ileum, liver, and spleen, along with that of the mucin gene, MUC2 (Fig. 7b) in the jejunum and ileum, are presented graphically.

***Effects of CP challenge***: CP challenge downregulated sIgA expression in jejunum (*P* = 0.02) and ileum (*P* = 0.0001), suggesting a possible reduction in mucosal secretory activity following CP challenge. Under challenge condition sIgA expression declined in liver (*P* = 0.0001) but not in spleen (*P* = 0.109). MUC2 expression was refractory to CP challenge in jejunum (*P* = 0.095) and ileum (*P* = 0.999).

***Effects of BV-OLS1101*:** Supplementation of BV-OLS1101upregulated sIgA expression in jejunum (*P* = 0.001), particularly in challenged birds although the dose response was not significant. A strong up regulatory effect (*P* = 0.0001) was also observed in ileum, liver, and spleen. BV-OLS1101supplementation influenced MUC2 expression in both the jejunum (*P* = 0.0001) and ileum (*P* = 0.001), with a dose-dependent trend apparent in the former.

***Interaction effects*:** A significant CP challenge*BV-OLS1101 interaction in jejunum (*P* = 0.0001) indicated that sIgA expression was augmented by BV-DFM, particularly under enteric challenge. Similar interactions were observed for sIgA expression in liver and spleen (*P* = 0.0001). For MUC2, a significant interaction was noted in the jejunum (*P* = 0.029) but not in ileum (*P* = 0.364).

## Discussion

The characterization of BV-OLS-1101 highlights its potential as a thermotolerant and functionally active DFM candidate for poultry. Its spores retained 89 % viability after exposure to 80°C for 60 minutes, demonstrating suitability for pelleted feed applications, where elevated temperatures often impair fewer stable strains (Amerah et al., 2013). The strain's ability to revert to its vegetative form and achieve high CFU counts under favorable conditions indicates strong biological activity necessary for persistence and functionality in the poultry gut. Survival under acidic and bile conditions further strengthens the probiotic profile of BV-OLS1101. The bacterium could maintain viability across a broad pH spectrum, including pH 2.0, a level representative of avian gastric environments (Clavijo et al., 2018). Its bile tolerance followed a similar trend, with higher concentrations resulting in moderate viability loss, consistent with physiological stress responses in the small intestine. These attributes suggest that the strain can survive initial digestive barriers and remain metabolically active in the GIT. In addition to its survival traits, BV-OLS-1101 exhibited dual functional properties related to pathogen inhibition and enzyme production. It showed sensitivity to most antibiotics tested, minimizing concerns regarding dissemination of AMR genes to the host. Its antagonistic activity against CP *in vitro* suggests its possible potential in reducing pathogen load *in vivo*, either through direct inhibition or competitive exclusion. This is particularly relevant in antibiotic-free poultry systems where natural microbiota modulation is critical. The strain also demonstrated extracellular protease activity, confirmed by clear zones on skim milk agar and azocasein hydrolysis. The data generated hitherto suggests that the strain can be a potential producer of subtilisin family of proteases, which are known for their antimicrobial, immunomodulatory, and digestive functions in poultry (Ojha et al., 2019; Cupi et al., 2022; Azrin et al., 2022). It is quite possible that owing to its strong proteolytic properties, the subtilisin groups of proteases may facilitate the process of degrading undigested feed particles and antinutritional factors. In this way they may completement the functions of endogenous proteases and segment intestinal health while supporting nutrient absorption. Taken together, BV-OLS1101 demonstrates favorable traits for probiotic use, including resilience during feed processing, tolerance to gastrointestinal stressors, antimicrobial potential, and proteolytic activity. These attributes align well with the functional demands of modern poultry production systems, particularly those seeking antibiotic-free alternatives.

Following the *in vitro* characterization, the in vivo findings further supported the functional potential of BV-OLS-1101 as a novel probiotic. Its dietary supplementation modulated the immune responses in the CC group which is reflected in the altered expression of key cytokines such as IL-1β, IFN-γ, IL-6 and TGF-β, along with the upregulation of mucosal immune markers, MUC2 and sIgA. These immunological shifts may be attributed, at least partially, to the subtilisin-like serine proteases secreted by BV-OLS-1101. Although the precise cellular mechanisms require further elucidation, earlier studies suggest that subtilisin-producing *Bacillus* strains can interact with gut-associated lymphoid tissues and contribute to enhanced mucosal immunity (Xu et al., 2021). Taken together, the results from this study position BV-OLS-1101 as a promising probiotic candidate. Its thermal resilience, antimicrobial potential, proteolytic activity, and immunomodulatory influence make it a suitable candidate for inclusion in poultry feed formulations perhaps as a replacement for the conventional AGPs.

To date, there are no published reports directly investigating the effects of purified subtilisin in broiler chickens subjected to CP challenge. However, several studies have examined the impact of BS strains known or likely to produce subtilisin. For example, Liu et al. (2021) demonstrated that dietary inclusion of *B. subtilis* PB6 improved intestinal morphology and microbial balance in CP-challenged broilers. Jayaraman et al. (2013) similarly reported reduced lesion scores, and pathogen load following BS supplementation. In a more recent study, showed that *B. subtilis* DSM29784 alleviated CP-induced gut damage by modulating the cecal microbiota and associated metabolic pathways (Wang et al., 2023). Although the specific role of subtilisin, which was not characterized in these studies, the observed benefits align with the known bioactivity of subtilisin which include its potential to modulate host immunity, suppress enteric pathogens leading to augmented performance as has been demonstrated in the present experiment.

The performance traits of the experimental chickens in this study, particularly the changes in FCR, reflect the broader immune-physiological landscape shaped by CP challenge and BV-DFM intervention. Although CP challenge did not significantly affect BW or ADG, the post-challenge impairment in FCR in the CC group indicated towards a biologically relevant disruption in nutrient utilization which was likely originated from the subclinical inflammation and mucosal stress following exposure to CP challenge. This hypothesis gets bolstered by the elevated expression of IL-1β, IL-6, TNF-α, and IFN-γ in the CC group. These shifts in the cytokine profile might compromise with the epithelial functioning of the infected birds leading to partitioning of metabolic resources more towards immune activation, thereby limiting growth efficiency. The improvement in FCR observed with BV-DFM supplementation, particularly in the CC groups, suggests a partial restoration of gut functionality. This benefit, despite the lack of significant increases in BW or ADFI, likely suggested an enhanced mucosal resilience, as indicated by upregulation of MUC2, sIgA, and TGF-β. These factors are known to strengthen epithelial integrity and promote local immune tolerance, which together support more efficient nutrient absorption under enteric stress (Mountzouris et al., 2007; Kogut and Arsenault, 2016). In this experiment the BV-DFM acted more as a physiological stabilizer rather than a direct growth promoter, which might have augmented the adaptive capacity of the host under conditions of enteric challenges. It is intriguing that the BV-DFM did not exert a pronounced anabolic effect in the non-challenged birds which reinforce the concept that the benefits of a gut acting growth promoter become most discernible only under conditions of intestinal perturbation (Bedford, 2000). This context-dependent efficacy aligns with the principle of host–microbiota crosstalk, wherein probiotic benefits are amplified during dysbiosis or inflammation (Ng et al., 2009). Furthermore, The dose-responsive trends observed with 12 × 10^8^ CFU/g which was equivalent to supplementation of 500 mg/kg of the BV OLS 1101containing premix demonstrated the need for adequate probiotic dosing to elicit measurable responses.

Liveability data supported the immunological effects associated with BV-DFM supplementation. Although statistical differences in mortality were not detected, birds supplemented with BV-DFM under CP challenge exhibited numerically higher survival rates during the early post-challenge phase. This time point almost overlapped with the peak inflammatory gene expression and lesion development, suggesting a modest yet biologically relevant protective effect of BV-DFM. Also, this trend plausibly indicates towards an early mucosal immune modulation and epithelial stabilization, rather than an overt disease suppression. Similar findings have been reported where inclusion of probiotic in dietary regime reduced mortality during enteric challenges by reinforcing mucosal immune competence and microbial balance (Shini et al., 2020; Brisbin et al., 2010).The statistical insignificance observed with regard to the gross lesion scores should be interpreted with caution, because the overall lesion severity was low and the classical scoring methods might not be competent enough to capture the subtle changes happening on the mucosal surface. Notably, BV-DFM supplementation, especially in presence of CP challenge, upregulated the expression of sIgA and MUC2 which are not only central to mucosal defence and microbial regulation in the GIT but also an indicator of an enhanced immune readiness of the gut to combat with an impending infection (Macpherson et al., 2008; Kim and Ho, 2010). These responses correlate well with the visibly healthier intestinal mucosa observed in the BV-DFM supplemented birds, as evidenced by the improved gross appearance in macroscopic photographs. The immunomodulatory effect observed here may be partly linked to the subtilisin-like proteases produced by BV-OLS-1101 (Xu et al., 2021). Subtilisin has been reported to possess both antimicrobial and immunoregulatory properties, including modulation of Toll-like receptor signaling and epithelial repair processes (Xu et al., 2021). While gross lesion attenuation was not evident, the transcriptional response indicates a strengthening of mucosal barrier integrity, potentially mediated by subtilisin-induced immune signaling.

Overall, the findings of this study demonstrated that BV-OLS-1101 exhibited several probiotic attributes of relevance to poultry production, including thermostability, antimicrobial activity, and the ability to produce subtilisin-like extracellular proteases. When supplemented as a DFM in broiler diets, BV-OLS-1101 improved FCR, particularly during the post-challenge period following induction of CP challenge, with improvements of up to 7.9 % compared to the challenged control group. While no significant changes in BW gain or ADFI were observed, the improvement in FCR indicates enhanced nutrient utilization efficiency, likely mediated by immunological stabilization and epithelial support.

It was concluded from the present study that at a dietary concentration of 6 -12 × 10^8^ CFU/g feed BV-OLS-1101, a thermostable, subtilisin-producing probiotic strain, improved FCR by up to 7.9 % following a CP challenge. These performance benefits were mediated by modulation of intestinal immune responses, including reduced expression of pro-inflammatory cytokines and upregulation of protective mucosal markers such as sIgA and MUC2. Although NE lesion scores remained unchanged, a visibly healthier gut morphology were noted in the supplemented birds. The effects of the probiotic were most evident under enteric stress, suggesting that its functional efficacy is primarily evident under challenge conditions when the DFM could confer greater immune protection and epithelial stability. With demonstrated antibiotic sensitivity and without apparent AMR risks, BV-OLS-1101 seems to be a promising candidate to augment performance, and sustainability in AGP -free poultry production systems.

## CRediT authorship contribution statement

**S. Haldar:** Conceptualization, Investigation, Supervision, Writing – review & editing. **A.K. Dhara:** Project administration. **I. Sengupta:** Methodology, Writing – original draft. **S. Paul:** Formal analysis, Writing – review & editing. **S.S. Arora:** Formal analysis. **R. Kumar:** Formal analysis. **A. Pal:** Formal analysis. **V. Kulkarni:** Funding acquisition.

## Disclosures

The authors declare that there is no competing interest.
